# Deconstruction of a spino-brain-spinal cord circuit drives chronic mechanical pain

**DOI:** 10.21203/rs.3.rs-5292927/v1

**Published:** 2025-10-13

**Authors:** Qian Wang, Joo-Han Lee, Gregory Nachtrab, Yuan Yuan, Lei Yuan, Wei Qi, Manuel Alexander Mohr, Jing Xiong, Mark A Horowitz, Xiaoke Chen

**Affiliations:** 1 Department of Biology, Stanford University; Stanford, California 94305, USA; 2 Department of Electrical Engineering, Stanford University; Stanford, CA 94305, USA.; 3 Equal contributions

## Abstract

Inflammation or nerve injury at periphery can cause chronic pain. Although the spinal cord-projecting neurons in the rostral ventromedial medulla (RVM^SC^ neurons) are known can promote pain chronification^1–4^, the pathway by which peripheral injury signals drive these neurons is poorly understood^5,6^. Here we report a circuit loop that extends from spinal cord to ventral posterolateral thalamus and posterior complex of the thalamus, proceeds to primary somatosensory cortex; then returns to the spinal cord via lateral superior colliculus, which in turn connects to μ-opioid receptor expressing RVM^SC^ neurons. Silencing any node along this multisynaptic circuit has minimal effect on nociception in healthy mice, but can eliminate mechanical hypersensitization and restore normal nociceptive response thresholds in mouse models of inflammatory and neuropathic pain. Repetitive, but not acute, activation of each node within this circuit in healthy mice is sufficient to cause robust chronic mechanical hypersensitization. Our findings elucidate a spino-brain-spinal circuit loop linking ascending and descending pathways that specifically drives chronic mechanical pain, and identify novel cellular targets for treating chronic pain.

Tissue inflammation or nerve injury typically results in temporary allodynia and hyperalgesia, which attracts attention to the affected area to prevent further damage and promote healing. Sometimes, this sensitization persists and leads to chronic pain, imposing tremendous psychological and socioeconomic burdens. While tissue damage often occurs at the periphery, accumulating evidence supports the crucial role of an endogenous pain modulation network in the central brain in the initiation and maintenance of chronic pain state^1–3^. The diffused brain regions in this network converge onto the periaqueductal gray (PAG)-rostral ventromedial medulla (RVM, including the raphe magnus and gigantocellular reticular nuclei) system, then interact with local pain circuitry in the spinal cord through RVM^SC^ neurons^1–6^. However, little is known how information about peripheral injury is transmitted to these descending RVM^SC^ neurons.

Classic *in vivo* electrophysiological studies have identified a group of RVM^SC^ neurons that are activated by nociceptive stimuli and inhibited by morphine, which is known as ON-cell^7–9^. The μ-opioid receptor (encoded by the *Oprm1* gene), the primary target of morphine, is a Gi-coupled G-protein coupled receptor whose activation suppresses neural activity. Therefore, these morphine-inhibited RVM^SC^ neurons should express OPRM1. Together, these response properties lead to a widely accepted working model hypothesizing that the activity of OPRM1^+^ RVM^SC^ neurons facilitates pain, potentially promoting chronic pain^1–3^. However, selective activation of OPRM1^+^ RVM^SC^ neurons in behaving animals has not been done, so this model has not been directly tested^4^. Moreover, if the model is correct, then how PAG inputs modulate the activity of these neurons becomes an interesting puzzle, as the net effect of PAG activation is reducing rather than promoting pain^10–12^.

Here we first developed new genetic and viral tools to access OPRM1^+^ RVM^SC^ neurons and demonstrated their specific role in driving pain sensitization after injury but not in acute pain. Using OPRM1^+^ RVM^SC^ neurons as a starting point, and combining monosynaptic retrograde tracing with pathway manipulation, we then mapped out a multisynaptic circuit, from the spinal cord to the primary somatosensory cortex through the spinothalamic tract, then returning to the spinal cord from the cortex through the OPRM1^+^ RVM^SC^ neurons via the lateral superior colliculus, but not the PAG. This circuit loop specifically involves in injury-caused mechanical hypersensitization but has limited contribution to acute nociceptive pain in healthy conditions, substantiates the potential of targeting this circuit for treating chronic pain while sparing protective nociceptive pain.

## Accessing OPRM1^+^ RVM^SC^ neurons

**Fig. 1:**
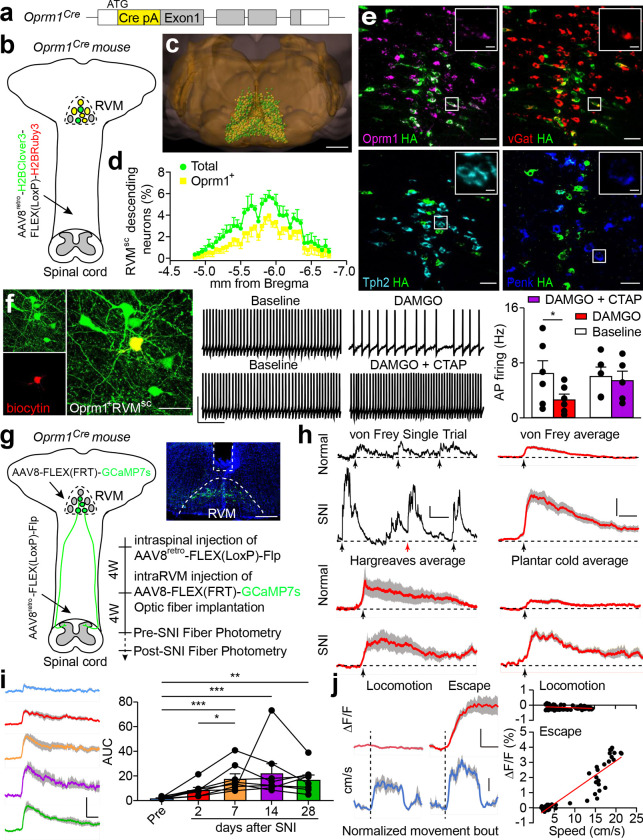
Labeling and recording of OPRM1^+^ RVM^SC^ neurons. **a**, Design of *Oprm1-Cre* knockin mice. **b**, Schematic shows spinal injection of AAV8^retro^-H2BClover3-FLEX(LoxP)-H2BRuby3 at P1.5 in Oprm1-Cre mice. **c**, 3D reconstruction of brainstem shows retrogradely labeled OPRM1^+^ (yellow) and OPRM1^−^ (green) RVM^SC^ neurons. Scale Bar: 1 mm. **d,** Distribution of RVM^SC^ neurons along A-P axis (n = 4). **e**, Example images of molecular characterization of OPRM1^+^ RVM^SC^ neurons (n = 5–8 slides from 4 mice). OPRM1^+^ RVM^SC^ neurons were retrogradely labeled with spinal injection of AAV8^retro^-FLEX(LoxP)-Rpl22–3XHA. RNAscope probes were used to visualize Oprm1 (magenta), vGat (red), Tryptophan Hydroxylase 2 (Tph2, cyan) and Proenkphalin (Penk, blue). HA tag (green) was visualized by immunostaining. Scale Bar: 50 μm. Insert, high magnification image of a representative neuron. Scale Bar: 10μm. See quantification in Extended Data Fig. 3d. **f**, Inhibition of OPRM1^+^ RVM^SC^ neurons by DAMGO. Left, representative image of a recorded OPRM1^+^ RVM^SC^ neuron (green) labeled by biocytin (red). Scale bar: 50 μm; Example traces (middle) and quantification (right) of action potential firing from recorded neurons before and after perfusion of DAMGO (1 μM, red, n = 6) or DAMGO+CTAP (1 μM + 1 μM, purple, n = 5). Scale bar: 50 mV, 1s. Wilcoxon signed-rank test, * *P* < 0.05. **g**, Experimental timeline for (**h), (i), (j**). W, week. Representative image shows fiber track and jGCaMP7s expression. Scale Bar: 500 μm. **h,** Example calcium traces of OPRM1^+^ RVM^SC^ neurons respond to mechanical (von Frey) and thermal (Hargreaves and Plantar cold) stimuli in normal and SNI mice. Single trial traces are in black, mean of averaged traces are in Red, and shade area indicates SEM (n = 7–8). Black arrow indicates time of paw withdrawal caused by stimuli. Red arrow indicates time of spontaneous paw withdrawal. Scale Bar: 5 s, 1% ΔF/F for single trial trace; 2 s, 1% ΔF/F for averaged traces. See quantification in Extended Data Fig. 4c. **i**, Representative traces (left) and quantification of area under curve (AUC, right) in OPRM1^+^ RVM^SC^ neurons to mechanical stimuli before SNI and 2,7,14,28 days after SNI (n = 8). Non-parametric ANOVA test, * *P* < 0.05, ** *P* < 0.01, *** *P* < 0.001. Scale bar: 2 s, 2% ΔF/F. **j,** Representative traces of calcium response (left, upper panel) and movement speed (left, lower panel), and correlation of calcium response vs. movement speed (right) in SNI mice. Dash line indicates the time of movement start. Dot indicates the average speed calcium signal during each movement event. Red line: linear fitting of dots. R^2^= 0.087 or 0.845 for locomotion and escape, respectively. Scale bar: 50% of normalized movement bout, 2% ΔF/F, 5 cm/s. See data for the uninjured healthy mice in Extended Data Fig. 4f, g. Locomotion: spontaneous movement without stimulus; Escape: escape response after von Frey stimulus. Mean ± SEM.

To access the OPRM1^+^ RVM^SC^ neurons, we generated a knockin mouse line to express Cre recombinase from the endogenous *Oprm1* locus (Oprm1-Cre mice, Fig. 1a and Extended Data Fig. 1a-c, supplemental note)^13^. However, besides RVM^SC^ neurons, local interneurons or ascending neurons in the RVM could also expresses the μ-opioid receptor (Extended Data Fig. 1d). In order to gain projection-specific access to the RVM^SC^ neurons, we optimized a neonatal spinal virus injection procedure and achieved broad spreading of adeno-associated virus (AAV) in the dorsal horn between cervical and lumbar spinal cord (Extended Data Fig. 2a–c)^14^. We also developed a new retrograde AAV by inserting the decapeptide LADQDYTKTA between N590 and T591 of the AAV8 capsid (AAV8^retro^) (Extended Data Fig. 2d)^15^. Compared to AAV2^retro^, this modification led to about 2.3-fold and 3.5-fold increases in retrogradely labeled RVM^SC^ neurons and cortical-thalamic projecting neurons, respectively (Extended Data Fig. 2e-g). AAV8^retro^ also labeled twice as many neurons as co-injected chemical tracer CTB and did not transport anterogradely into postsynaptic neurons (Extended Data Fig. 2h,i). We injected AAV8^retro^-H2B-Clover3-FLEX(LoxP)-H2B-Ruby3 into the spinal cord of Oprm1-Cre mice at P1.5 to express nuclear-localized Ruby3 and Clover3 in OPRM1^+^ and all spinal projecting neurons, respectively (Fig. 1b). We labeled about 5600 RVM^SC^ neurons per mouse ranging from bregma −4.7 to −6.5 mm, 65% of which express the μ-opioid receptor and these OPRM1^+^ RVM^SC^ neurons located mainly in the raphe magnus (Fig. 1c,d, Extended Data Fig. 2j)^16^. Using Transparent Embedding Solvent System (TESOS) method and single axon tracing^17^, we found that many of these neurons traveled together in the lateral funiculus before they enter into the spinal cord from the deep laminar, then branches into thin axon terminals to innervate the superficial laminar of the dorsal horn, suggesting its potential role in modulating somatosensation (Extended Data Fig. 3a-c)^17^. The majority of OPRM1^+^ RVM^SC^ neurons are GABAergic, consistent with previous observation (Fig. 1e, Extended Data Fig. 3d)^18^. Although the OPRM1^+^ RVM^SC^ neurons are not serotoninergic, intraspinal injection of AAV8^retro^ can robustly label descending serotoninergic neurons concentrated in the median raphe and in lateral paragigantocellularis (Fig. 1e, Extended Data Fig. 3d,e). Action potential firing can be inhibited by DAMGO, a highly selective peptide agonist for μ-opioid receptor, confirming the expression of functional μ-opioid receptor in OPRM1^+^ RVM^SC^ neurons (Fig. 1f). Proenkephalin (Penk) is expressed in a group of GABAergic RVM^SC^ neurons that can form directly axo-axonic interaction with peripheral sensory inputs from dorsal root ganglion^19^. Interestingly, most OPRM1^+^ RVM^SC^ neurons do not express Penk, suggesting that at least two distinct descending GABAergic pathways from RVM could modulate sensory processing in the spinal cord (Fig. 1e and Extended Data Fig. 3d).

## Noxious stimuli activate OPRM1^+^ RVM^SC^ neurons

Besides the RVM, many neurons in the locus coeruleus (LC) were also retrogradely labeled (Extended Data Fig. 3f), consistent with robust *Oprm1* expression in LC^20^. We therefore devised an intersectional strategy to specifically target OPRM1^+^ RVM^SC^ neurons while sparing OPRM1^+^ LC^SC^ neurons^21^. We first injected AAV8^retro^ expressing Cre-dependent Flp (AAV8^retro^-FLEX(LoxP)-Flp) into the spinal cord of Oprm1-Cre mice at P1.5 to express Flp recombinase in all OPRM1^+^ spinal cord-projecting neurons, including both LC and RVM. Three to six weeks later, we injected AAV8 expressing Flp-dependent effectors (AAV8-FLEX(FRT)-effectors) into the RVM of these mice to achieve restricted expression of various effectors in OPRM1^+^ RVM^SC^ neurons. Using this strategy, we transduced OPRM1^+^ RVM^SC^ neurons with genetically encoded neuronal activity sensor jGCaMP7s^22^, then used fiber photometry to record their *in vivo* activity during von Frey, Hargreaves and plantar cold tests (Fig. 1g)^23–27^. In healthy mice, these neurons exhibited calcium transients time-locked to paw withdrawal in response to all three types of noxious stimuli (Fig.1h, Extended Data Fig. 4a, b). Following spared nerve injury (SNI) to induce a severe and persistent neuropathic pain state^28^, these neurons displayed heightened responses to mechanical and cold, but not heat, stimuli (Fig.1h, Extended Data Fig. 4c). Calcium responses in the OPRM1^+^ RVM^SC^ neuron increased from day 2 to day 7 post-SNI and remained elevated (Fig.1i). In contrast, in a Complete Freund’s Adjuvant (CFA)-induced inflammatory pain model^29^, calcium responses peaked at day 2 before gradually returning to baseline over 21 days (Extended Data Fig. 4d, e). Notably, these neuron showed minimal activity during voluntary locomotion or in the absence of paw withdrawal, yet exhibited robust activity during pain-evoked behaviors (Fig.1h–j, Extended Data Fig. 4a,f,g)^30^. The amplitude of this activity correlated with the speed of the escape response (Fig.1j, Extended Data Fig. 4f, g) and was also observed during spontaneous pain episodes (e.g. paw flinching) at levels comparable to evoked responses (Fig. 1h, Extended Data Fig. 4h)^28^. Collectively, these observations indicate that OPRM1^+^ RVM^SC^ neurons are specifically activated by nociception, sensitized by peripheral injury, but not active during locomotion.

## OPRM1^+^ RVM^SC^ neurons mediate mechanical and cold hypersensitization

**Fig. 2:**
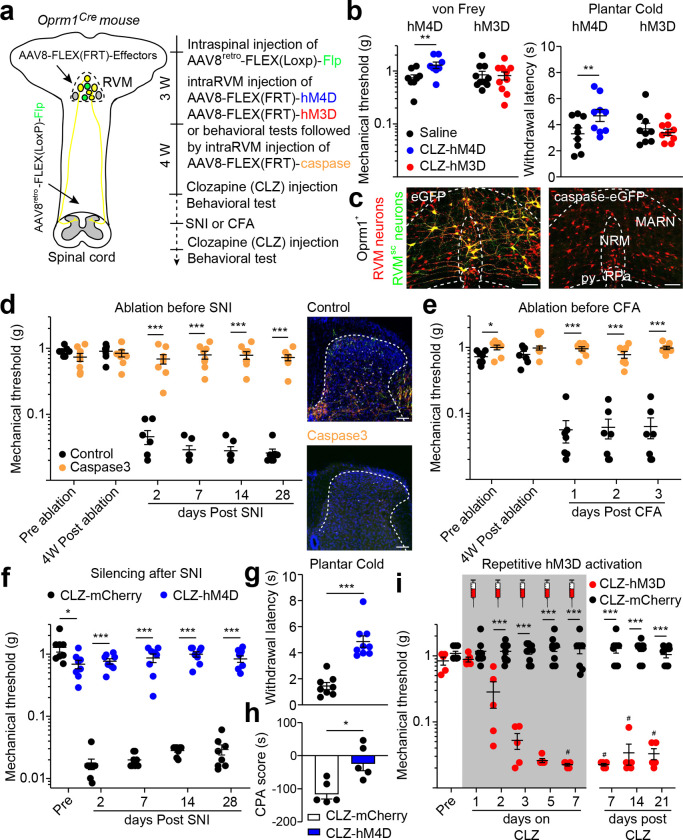
OPRM1^+^ RVM^SC^ neurons are required for initiation and maintenance of mechanical pain sensitization. **a**, Experimental timeline for (**b**), (**d**), (**e**), (**f**), (**g**). W, week. **b**, Quantification of saline (black) or CLZ on mechanical threshold (left) and plantar cold withdrawal latency (right) of hM4D (blue, n = 8–9) or hM3D (red, n = 9–10) expressing healthy mice. See Hargreaves test results in Extended Data Fig. 6a. CLZ: clozapine. Wilcoxon matched-pairs signed rank test, ** *P* < 0.01. **c**, Representative images of caspase caused ablation of OPRM1^+^ RVM^SC^ neurons. OPRM1^+^ RVM neurons are labeled with Ruby3 (red). OPRM1^+^ RVM^SC^ neurons express both Ruby3 and Clover3 (yellow, left). OPRM1^+^ RVM^SC^ neurons (yellow cells) were ablated by caspase expression (right). Dash lines indicate boundary of brain regions in the RVM. NRM: Nucleus raphe magnus, RPa: Nucleus raphe pallidus, MARN: Magnocellular reticular nucleus, py: pyramid. See quantification in Extended Data Fig. 7b. Scale Bar: 100 μm. **d**, Quantification of mechanical thresholds of control- (black, n = 7) and Caspase3- (orange, n = 8) expressing mice after SNI (left). Right: Representative image shows OPRM1^+^ RVM^SC^ neurons terminals (yellow) and mechanical stimuli evoked c-Fos signals (white) in control- (upper panel) but not Caspase3- (lower panel) expressing mice. Dash line indicates the outline of the dorsal horn. See quantification in Extended Data Fig. 7d. Scale Bar: 100 μm. Mann-Whitney test, *** *P* < 0.001. **e**, Quantification of mechanical thresholds of control- (black, n = 7) and Caspase3- (orange, n = 8) expressing mice after CFA injection. Mann-Whitney test, * *P* < 0.05; *** *P* < 0.001. **f**, Quantification of mechanical thresholds after CLZ injection in hM4D- (blue, n = 8) and mCherry- (black, n = 8) expressing mice after SNI. Mann-Whitney test, * *P* < 0.05; *** *P* < 0.001. **g**, Quantification of Plantar cold withdrawal latency after CLZ injection in hM4D- (blue, n = 9) and mCherry- (black, n = 8) expressing mice after SNI. Mann-Whitney test, *** *P* < 0.001. **h**, Quantification of mechanical stimuli (0.16 g von Frey filament) evoked CPA in control (black, n = 5) but not in hM4D (blue, n = 5) expressing mice after SNI. See example locomotor traces in Extended Data Fig. 6c. Mann-Whitney test, * *P* < 0.05. **i**, Quantification of mechanical thresholds after repetitive daily injection of CLZ in hM3D- (n = 5) and mCherry- (n = 9) expressing healthy mice. CLZ was injected 23 hours before each von Frey test for 7 consecutive days (shade area). Dunn’s multiple comparisons test, # *P* < 0.05 for hM3D-expressing mice comparing before vs. after repetitive CLZ infusion. Mann-Whitney test, *** *P* < 0.001 for comparison between hM3D- and mCherry- expressing mice. Mean ± SEM.

The ability to effectively access the OPRM1^+^ RVM^SC^ neurons offers the opportunity to determine their roles in normal nociception and pain sensitization caused by nerve injury and inflammation. We transduced OPRM1^+^ RVM^SC^ neurons with inhibitory or excitatory designed receptors hM4D or hM3D^31^, respectively, so that these neurons could be experimentally activated or inhibited by intraperitoneal (I.P.) injection of clozapine (Fig. 2a)^32^. Our results showed that acute chemogenetic inhibition or activation of OPRM1^+^ RVM^SC^ neurons in these mice had little effect on their behavioral responses to mechanical or temperature stimuli, or on their locomotion, indicating a small contribution of the descending OPRM1^+^ pathway to protective acute nociception and locomotor function (Fig. 2b, Extended Data Fig. 5a-c Extended Data Fig. 6a,b)^4,33^. This is different from activation of descending serotoninergic neurons in the RVM, in which single activation can cause prolonged hypersensitivity^34^.

We next investigated the roles of OPRM1^+^ RVM^SC^ neurons in neuropathic and inflammatory pain. We reasoned that if OPRM1^+^ RVM^SC^ neurons are required for developing persistent pain, then ablation of these neurons before injury should prevent its initiation. We expressed activated Caspase3 in OPRM1^+^ RVM^SC^ neurons to induce cell-autonomous apoptosis^35^, which eliminated their cell bodies in the RVM and their terminals in the spinal cord (Fig. 2c,d, Extended Data Fig. 7a,b). These mice have normal mechanical thresholds and nociceptive stimuli-induced c-Fos in the dorsal horn (Fig. 2c,d, Extended Data Fig. 7c), but neither SNI nor CFA injection decreased mechanical withdrawal thresholds in the von Frey test (Fig. 2d,e). Mechanical stimulation after injury no longer caused increased c-Fos expression in the dorsal horn of the spinal cord (Fig. 2d, Extended Data Fig. 7d). Notably, ablation of OPRM1^+^ RVM^SC^ neurons only have a partial effect on CFA induced thermal hypersensitivity (Extended Data Fig. 7e). Thus, OPRM1^+^ RVM^SC^ neurons are required for the initiation of SNI- and CFA-induced mechanical hypersensitization.

Similarly, if activity of OPRM1^+^ RVM^SC^ neurons is required for maintaining the persistent pain state, then silencing these neurons after injury should alleviate established pain. SNI produces a severe chronic neuropathic pain state. This model is characterized not only by somatosensory hypersensitivity, which drives heightened affective-motivational responses to evoked stimuli^28,36^, but also by spontaneous pain, which is triggered by the synchronized cluster firing of injured sensory neurons^37^. We again utilized chemogenetic silencing of OPRM1^+^ RVM^SC^ neurons and evaluated its impact on sensory hypersensitivity, affective behaviors, and spontaneous pain. We found that clozapine infusion completely reversed sensitized mechanical and cold pain thresholds to normal level in SNI mice expressing hM4D but not mCherry in OPRM1^+^ RVM^SC^ neurons (Fig. 2f,g). The same treatment also normalized stimulus-evoked affective behaviors, including paw attending and escape response, and blocked mechanical stimuli induced conditioned place aversion (CPA) (Fig. 2h, Extended Data Fig. 6c, Extended Data Fig.8a)^4,38^. We further quantified spontaneous pain by monitoring stimulus-independent episodes of licking and flinching of the injured paw, and by employing an automated facial grimacing scale^39^. SNI mice exhibited significantly elevated spontaneous pain behaviors and grimace scores, and silencing of OPRM1^+^ RVM^SC^ neurons reduced both of these indicators of spontaneous pain down to the levels seen in non-injured control animals (Extended Data Fig.8b,c). Together, these observations indicate the reduction of both sensory and affective components of pain after silencing of OPRM1^+^ RVM^SC^ neurons. Importantly, 28 days after SNI, the time point when SNI-induced mechanical hypersensitivity becomes morphine resistant^40^, silencing these neurons still robustly alleviated neuropathic pain (Fig. 2f). Moreover, examining c-Fos expression evoked by mechanical stimuli in multiple pain-related brain regions, we found that chemogenetic silencing of OPRM1^+^ RVM^SC^ neurons in SNI mice restored c-Fos expression to the level as the sham surgery mice, revealing a brain wide reduction of pain responses after silencing of these neurons (Extended Data Fig. 6d,e).

In contrast to the minimal effect on mechanical threshold after acute activation (Fig. 2b), repetitive chemogenetic activation of OPRM1^+^ RVM^SC^ neurons in healthy mice induced profound and long-lasting mechanical hypersensitivity and drove heightened stimulus-evoked affective behaviors (Fig. 2i, Extended Data Fig.8a). In addition, these mice also became sensitized to plantar cold stimulus but did not change its response to heat (Extended Data Fig. 6f,g). Together, these results reveal that the descending OPRM1^+^ pathway is necessary and sufficient for both the initiation and maintenance of mechanical hypersensitivity and cold allodynia^4,33^. Interestingly, these mice did not show more spontaneous licking and flinching or facial grimace than control, and also did not show generalized sensitization to air puff to the cornea (Extended Data Fig.8b-d).

Beyond sensory hypersensitivity, chronic pain is frequently comorbid with anxiety and depression, affecting approximately 40% of adult patients^41^. Consistent with this, we observed heightened anxiety- and depression-like states in SNI mice six weeks after surgery, evidenced by reduced center time in the open field test (OFT), less time in the open arm of the elevated plus maze (EPM), and increased immobility in the tail suspension test (TST, Extended Data Fig. 8e-g)^42^. Given that manipulating OPRM1^+^ RVM^SC^ neurons modulates both sensory and affective components of pain, we thus examined whether these neurons also contribute to the comorbid states. We found that repetitive chemogenetic activation of OPRM1^+^ RVM^SC^ neurons in healthy mice was sufficient to induce anxiety-like, but not depression-like, behaviors. In contrast, chemogenetic silencing of these neurons in SNI mice had no effect on the established anxiety- and depression-like states, suggesting that while their activity can drive anxiety, they are not required for maintaining these comorbid emotional symptoms after nerve injury (Extended Data Fig. 8e-g).

## Lateral superior colliculus input onto OPRM1^+^ RVM^SC^ neurons

**Fig. 3:**
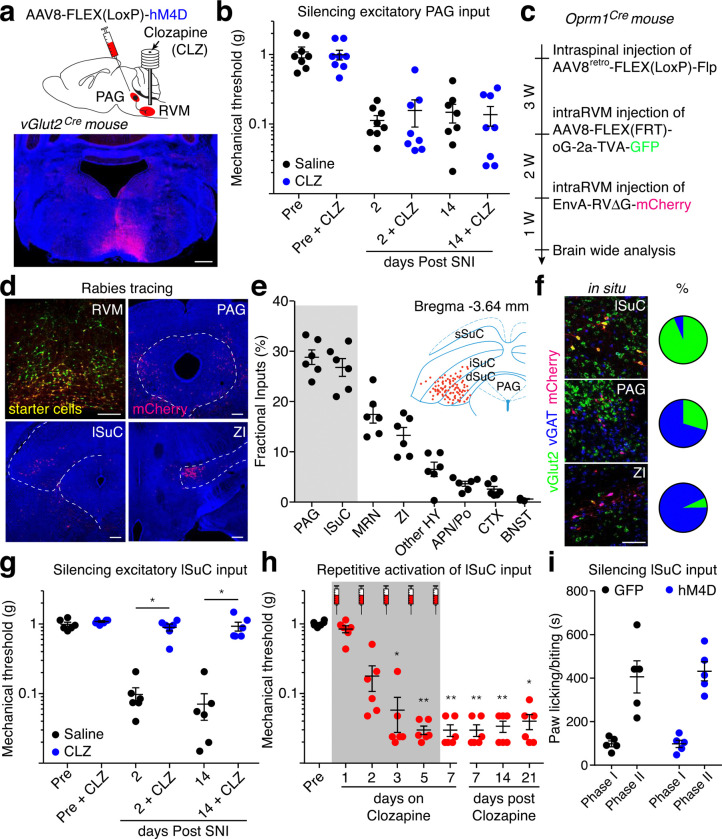
Lateral superior colliculus (lSuC) provides major excitatory inputs onto OPRM1^+^ RVM^SC^ neurons and drives mechanical pain sensitization. **a**, Schematic (upper panel) of chemogenetic silencing of the PAG→RVM pathway. Representative image shows vGlut2^+^ terminals (red) from PAG in the RVM. CLZ: clozapine. Scale Bar: 500 μm. **b**, Quantification of mechanical thresholds of saline (black) and CLZ (blue) infusion in the same group of hM4D expressing mice (n = 8). **c**, **d**, **e**, Experimental timeline (**c**)**,** representative images (**d**), and quantification (**e**) of monosynaptic inputs onto OPRM1^+^ RVM^SC^ neurons (n = 6). Scale Bar: 250 μm. Inset: mCherry^+^ neurons are concentrated in intermediate SuC (iSuC) and deep SuC (dSuC) but not superficial SuC (sSuC) at Bregma −3.64mm. PAG: periaqueductal gray; lSuC: lateral superior colliculus; MRN: midbrain reticular nucleus; ZI; zona incerta; HY: hypothalamus; APN: anterior pretectal nucleus; Po: posterior complex of thalamus; CTX: cortex; BNST: bed nucleus of the stria terminalis. **f**, Representative images (left) and quantification (right) of RNAscope staining of vGlut2 (green) and vGat (blue) in mCherry positive retrogradely labeled input neurons from lateral SuC (lSuC), PAG and Zona Incerta (ZI) (6 slides from 3 mice). Scale Bar: 50 μm. **g**, Quantification of mechanical thresholds of saline (black, n = 6) and CLZ (blue, n = 6) infusion into the RVM of vGlut2^Cre^ mice with hM4D expressing in lSuC for chemogenetic silencing of the lSuC→RVM pathway. Wilcoxon matched-pairs signed rank test, * *P* < 0.05. **h**, Quantification of mechanical thresholds after repetitive daily RVM infusion of CLZ in non-injured vGlut2^Cre^ mice with hM3D expressing in lSuC for chemogenetic activation of the lSuC→RVM pathway (n = 6). Clozapine was injected 23 hours before each von Frey test for 7 consecutive days (shade area). Dunn’s multiple comparisons test, * *P* < 0.05, ** *P* < 0.01. **i**, Quantification of time for paw licking and biting after formalin injection into the hindpaw. CLZ was locally infused into the RVM of vGlut2^Cre^ mice with eGFP- (black, n = 5) and hM4D- (blue, n = 5) expressing in lSuC for chemogenetic silencing of the excitatory lSuC→RVM pathway. Mean ± SEM.

We next used the OPRM1^+^ RVM^SC^ neuron as a starting point to map the neuronal circuits sending injury information from periphery onto this critical descending output for driving chronic mechanical pain. Since peripheral stimuli strongly activate OPRM1^+^ RVM^SC^ neurons (Fig. 1h), we thus focused on identifying the excitatory monosynaptic inputs onto these neurons that drives chronic pain. Current models emphasize PAG to RVM pathway in descending pain modulation^1–3^. We therefore first examined the impact of silencing the excitatory PAG terminals in the RVM on nociception and mechanical hypersensitivity. We injected AAV8-FLEX(LoxP)-hM4D into the ventrolateral PAG of vGlut2-Cre mice, then infused clozapine in the RVM to silence the excitatory PAG→RVM pathway, which is validated using ex-vivo slice recording (Fig. 3a, Extended Data Fig. 5d-f, Extended Data Fig. 11a,b). Interestingly, chemogenetic silencing of this pathway had no effect on either the baseline mechanical threshold or SNI-induced mechanical hypersensitivity (Fig. 3b), consistent with the predominate analgesic effect of PAG stimulation and indicates the existence of other excitatory inputs responsible for driving the engagement of OPRM1^+^ RVM^SC^ neurons in mechanical hypersensitivity^12,43^.

We then performed cTRIO (cell-type specific Tracing the Relationship of Inputs and Outputs) experiments to reveal all monosynaptic inputs onto OPRM1^+^ RVM^SC^ neurons^18,21^. We expressed codon-optimized glycoprotein and EnvA receptor specifically in the OPRM1^+^ RVM^SC^ neurons, then injected EnvA-pseudotyped mCherry expressing G-deleted rabies virus into the RVM (Fig. 3c)^44,45^. We then examined mCherry expressing neurons throughout the entire brain. Notably, we found that intermediate/deep gray layers of lateral superior colliculus (lSuC, ranging from bregma −3.45 to −3.65 mm) contained a similar number of retrogradely labeled neurons as in PAG (Fig. 3d,e)^46^. Our cTRIO tracing only labeled very few neurons in the lateral parabrachial nuclei (lPBN), which has recently been shown provided direct inputs onto ON-cells in the RVM^5,47^. In contrast, cTRIO tracing from the entire population of GABAergic RVM^SC^ neurons revealed input from PBN^18^. This discrepancy could reflect the tropism of the rabies virus, or because lPBN neurons prefer to innervate local ON-cells or OPRM1^−^ RVM^SC^ neurons but not to OPRM1^+^ RVM^SC^ neurons^47,48^. We further examined these brain regions using RNAscope probing for inhibitory (VGAT) and excitatory (VGLUT2) neurons and found that about 30% of retrogradely labeled neurons in PAG were VGLUT2^+^, whereas this number was more than 90% in lSuC (Fig. 3f). Therefore, lSuC provides more excitatory input onto OPRM1^+^ RVM^SC^ neurons than PAG.

In consistent with its robust anatomical connection, functional connection between lSuC and RVM is essential for mechanical hypersensitization. The excitatory RVM projecting neurons in lSuC showed significantly larger response to von Frey stimuli after SNI (Extended Data Fig. 4i-k). We chemogenetically silenced excitatory lSuC terminals in RVM after SNI and found it restored normal mechanical thresholds (Fig. 3g, Extended Data Fig. 11a,b) and eliminated pain-induced CPA in SNI mice (Extended Data Fig. 9a), but had no effect on locomotion (Extended Data Fig. 5g). Similarly, silencing of this pathway also eliminated CFA-induced mechanical but not thermal sensitization, indicated its important role in inflammatory pain (Extended Data Fig. 6h,i). A previous study reported that lSuC is part of orienting circuits for defensive response and lSuC lesion prevented licking and biting towards the paw injected with formalin^49^. It is therefore possible that the ISuC→RVM pathway is part of this defensive circuit and silencing this pathway would make the animal less attentive to their pain. To directly examine this possibility, we chemogenetically silenced the excitatory ISuC→RVM pathway in formalin injected mice and found no effect on either licking and biting towards formalin-injected paw (Fig. 3i). Furthermore, optogenetic activation of this pathway also had no effect on locomotion, turning, heart and breathing rate (Extended Data Fig. 5h-k).

Lastly, repetitive chemogenetic activation of excitatory lSuC inputs in the RVM caused long-lasting mechanical hypersensitization and pain-induced CPA in non-injured healthy mice (Fig. 3h, Extended Data Fig. 9b, 11a,b). Together, these results established a causal role of the excitatory lSuC→RVM pathway in driving mechanical hypersensitivity.

## SS drives chronic mechanical pain though lSuC

**Fig. 4:**
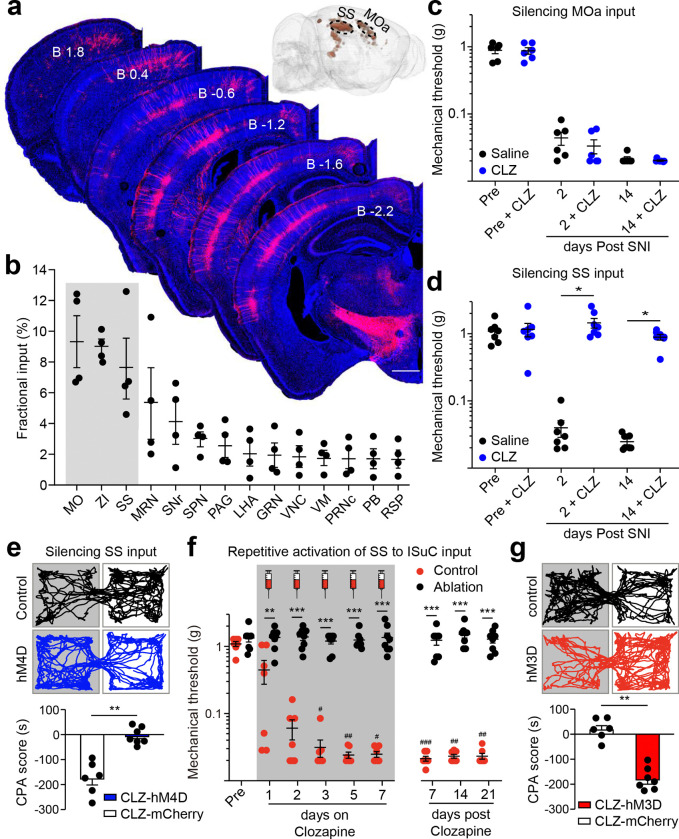
Somatosensory cortex (SS) to lSuC pathway drives mechanical hypersensitization. **a, b,** Representative images (**a**) and quantification (**b**) of monosynaptic inputs onto RVM projecting neurons in the lSuC (n = 4). Scale Bar: 500 μm. Inset: reconstruction of retrogradely labeled neurons in the cortex. Dashed circle highlights the anterior motor cortex (MOa) and SS. MO: motor cortex; ZI: zona incerta; SS: somatosensory cortex; MRN: midbrain reticular nucleus; SNr: substantia nigra; APN: anterior pretectal nucleus; PAG: periaqueductal gray; LHA: lateral hypothalamic area; GRN: gigantocellular reticular nucleus; VNC: vestibular nuclei; VM: ventral medial nucleus of the thalamus; PRNc: pontine reticular nucleus, caudal part; PB: parabrachial nucleus; RSP: retrosplenial area. **c, d,** Quantification of mechanical thresholds of saline (black, n = 6–7) and CLZ (blue, n = 6–7) infusion into the lSuC of mice expressing hM4D in MOa (**c**) or SS (**d**) for chemogenetic silencing of the MOa→lSuC pathway or SS→lSuC pathway, respectively. CLZ: clozapine. Wilcoxon matched-pairs signed rank test, * *P* < 0.05. **e,** Example traces and quantification of mechanical stimuli (0.16 g von Frey filament) evoked CPA in mCherry (white, n = 6) but not hM4D (blue, n = 7) expressed in SS after SNI. Mann-Whitney test, ** *P* < 0.01. **f,** Quantification of mechanical thresholds of non-injured mice expressing hM3D in SS after repetitive CLZ infusion into the lSuC in control mice (red, n = 7) or mice after ablation of OPRM1^+^ RVM^SC^ neurons (black, n = 8). CLZ was injected 23 hours before each von Frey test for 7 consecutive days (shade area). Comparing control vs. ablation: Mann-Whitney test, ** *P* < 0.01, *** *P* < 0.001; Comparing before vs. after CLZ: Dunn’s multiple comparisons test, # *P* < 0.05, ## *P* < 0.01, ### *P* < 0.001. **g,** Example traces and quantification of mechanical stimuli (0.16 g von Frey filament) evoked CPA in hM3D- (red, n = 7) but not in mCherry- (white, n = 6) expressing mice after repetitive infusion of CLZ into the lSuC. Mann-Whitney test, ** *P* < 0.01. Mean ± SEM.

What are the upstream inputs onto lSuC that drive chronic mechanical pain? We again employed the cTRIO approach to map brain-wide monosynaptic inputs onto the RVM-projecting excitatory lSuC neurons. This revealed two prominent clusters of retrogradely labeled neurons in the cortex, located in the layer 5 of the somatosensory cortex (SS) and the anterior motor cortex (MOa) (Fig. 4a,b). Retrogradely labeled lSuC-projecting SS neurons concentrated between bregma −1.2 to −2.2 mm, whereas the retrogradely labeled MOa neurons were located within the anterior cortex region identified by retrograde labeling from lateral rostral medulla, which collaterally innervate the same lSuC regions that project to the RVM^50^ (Fig. 4a inset). Chemogenetic silencing the SS→ISuC, but not the MOa→ISuC, pathway eliminated SNI-induced mechanical hypersensitivity and CPA (Fig. 4c–e, Extended Data Fig. 11c,d), whereas repetitive chemogenetic activation of the SS→ISuC pathway induced chronic mechanical pain and pain-induced aversion in healthy mice (Fig. 4f,g, Extended Data Fig. 11c,d). Notably, this same activation protocol failed to drive mechanical hypersensitization in mice where OPRM1^+^ RVM^SC^ neurons had been ablated, highlighting that an intact descending pathway from SS to spinal cord is required for pain sensitization (Fig. 4f). Together, these findings extended the descending pathway for chronic mechanical pain one synapse upstream, from lSuC→RVM→spinal cord to SS→lSuC→RVM→spinal cord^51^. The fact that somatosensory cortex drives the lSuC→RVM pathway for chronic pain also reveals the anatomical and functional complexity in the superior colliculus.

## Parallel spinothalamic pathways for chronic mechanical pain

**Fig. 5:**
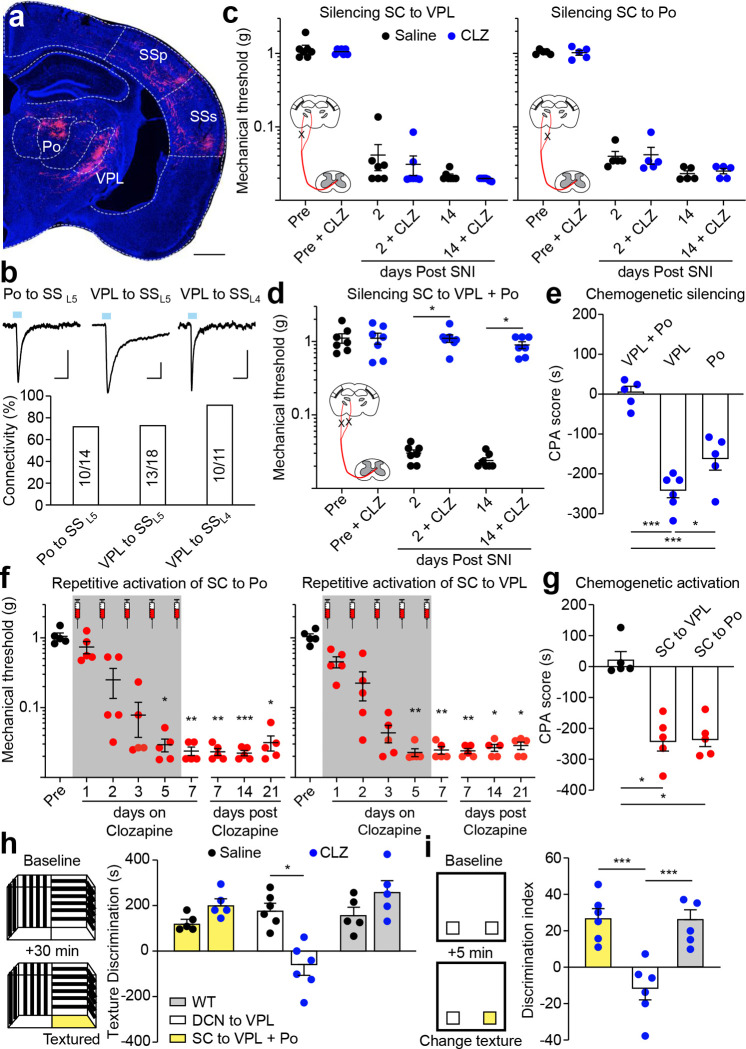
Parallel spinothalamic tract (STT) pathways drive mechanical hypersensitization. **a,** Representative images of transsynaptically labeled neurons in the thalamus and their terminals in the SS. Transsynaptic AAV1-Cre was injected in the spinal cord (SC), and AAV8-FLEX-mScarlet was injected in the thalamus. Po: posterior complex of the thalamus; VPL: ventral posterolateral nucleus. Scale Bar: 500 μm. **b,** Functional connectivity between Po, VPL with retrogradely labeled lSuC projecting neurons in layer 5 and neighboring layer 4 neurons in SS. Upper panel: representative traces of optical EPSC (oEPSC). Lower panel: Quantification of connectivity. Number in bar graph: cell with oEPSC/total recorded cells. Scale bar: 50 ms, 50 pA. **c,** Quantification of mechanical thresholds after saline (black, n = 5–7) and CLZ (blue, n = 5–7) injection into mice expressing hM4D in the SC→VPL pathway (left) or SC→Po pathway (right), respectively. CLZ: clozapine. **d,** Quantification of mechanical thresholds after saline (black, n = 7) and CLZ (blue, n = 7) injection into mice expressing hM4D in both the SC-VPL and SC-Po pathways. Wilcoxon matched-pairs signed rank test, * *P* < 0.05. **e,** Quantification of CPA after silencing SC-VPL (n = 6) or SC-Po (n = 5) pathway along or SC→VPL + SC→Po pathways together (n = 5). One-way ANOVA, * *P* < 0.05, *** *P* < 0.001. **f,** Quantification of mechanical thresholds of non-injured mice after repetitive CLZ injection into mice expressing hM3D in SC→Po (left, n = 5) or SC→VPL (right, n = 5). Black, before CLZ infusion; red, after CLZ infusion. Mechanical thresholds: Dunn’s multiple comparisons test, * *P* < 0.05, ** *P* < 0.01, *** *P* < 0.001. **g,** Quantification of CPA after repetitive activation of SC-VPL (n = 5) or SC-Po (n = 5) pathway. Control (Black, n = 5). Non-parametric ANOVA, * *P* < 0.05. **h,** Diagram (left) and quantification (right) of silencing the SC→VPL + SC→Po pathways together (yellow, n = 5) or the DCN-VPL pathway (white, n = 6) or wildtype mice without manipulation (WT, gray, n = 5) on texture discrimination score. Black, saline; blue, CLZ. Wilcoxon matched-pairs signed rank test, * *P* <0.05. **i,** Diagram (left) and quantification (right) of silencing the SC→VPL + SC→Po pathways together (yellow, n = 6) or the DCN→VPL pathway (white, n = 6) or wildtype mice without manipulation (WT, gray, n = 5) on novel object with novel texture. One-way ANOVA, *** *P* < 0.001. Mean ± SEM.

The spinothalamic tract (STT) has traditionally been considered as the major ascending pathway that transmitting innocuous sensory discriminative information and pain from the peripheral to the SS via the thalamus^52–54^. The prominent target of the STT in the thalamus is the ventral posterolateral nucleus (VPL), with some minor innervation of other thalamic nuclei, including the posterior complex of the thalamus (Po)^52–54^. The Po→SS pathway is involved in injury-induced pain sensitization but whether this function requires input from STT is not known^55^. Moreover, besides inputs from the spinal cord, both VPL and Po also relay other somatosensory inputs to SS^56,57^. We thus sought to selectively manipulate the VPL→SS and Po→SS pathway that convey information from the STT (STT→VPL→SS; STT→Po→SS). To achieve that, we leveraged the monosynaptic anterograde transsynaptic traveling property of the high-titer AAV1-Cre^58^. We injected AAV1-Cre into the lumbar region of the spinal cord dorsal horn and AAV8-FLEx-hM4D into the VPL, Po, or both, so that hM4D will only be expressed in VPL or Po neurons that receives STT inputs (Fig. 5a). Electrophysiological recording of retrogradely labeled lSuC projecting neurons in the layer 5 of SS (SS_L5_) revealed robust functional connectivity in both VPL→SS_L5_ and Po→SS_L5_ pathways (Fig. 5b). We next injected clozapine to selectively silence the STT→VPL→SS, STT→Po→SS, or both. We found that chemogenetic silencing of either pathway along had no effect on mechanical hypersensitization after SNI and pain-induced CPA (Fig. 5c,e, Extended Data Fig. 9c). Instead, chemogenetic silencing both pathways together abolished SNI-induced chronic mechanical pain and CPA (Fig. 5d,e, Extended Data Fig. 9c). These observations suggest that either STT→VPL→SS or STT→Po→SS pathway is sufficient to drive mechanical hypersensitization after injury. Indeed, repetitive chemogenetic activation of either pathway drove chronic mechanical pain in healthy mice and this pain sensitization effect also required the OPRM1^+^ RVM^SC^ neurons (Fig. 5f,g, Extended Data Fig. 9d, 10a,b). Lastly, six weeks after repetitive chemogenetic activation of the STT pathway, the mice showed heightened anxiety state but not depression-like behavior, similar to the activation of OPRM1^+^ RVM^SC^ neurons (Extended Data Fig. 8e,f,g, 10c,d). Together, we found two parallel thalamic pathways connecting the STT to the SS for chronic mechanical pain.

Chemogenetic silencing of either the STT→VPL→SS or STT→Po→SS pathway separately or simultaneously showed no effect on nociceptive thresholds before SNI, indicating that neither pathway is involved in nociception in healthy mice (Fig. 5c,d, Pre, Pre + CLZ). Since traditional view also suggest a potential role of STT in somatosensory discrimination, we thus further examined contribution of these pathways to behaviors requiring discrimination of innocuous tactile stimuli. Using a behavioral chamber with two compartments that have distinct floor textures (smooth Plexiglass vs. fine grit sandpaper), we found freely moving mice spent significant more time in the compartment with the sandpaper floor (Fig. 5h). We also performed a texture-based novel object recognition test and found that mice spent more time examining objects with novel textures (Fig. 5i)^59^. Silencing both the STT→VPL→SS and STT→Po→SS pathways together had no effect on mice’s preference for the sand paper floor or to objects with novel textures (Fig. 5h,i). These results reveal the limited contribution of the STT→thalamus→SS pathway in tactile discrimination, further supporting its selective role in mediating chronic mechanical pain after injury.

The VPL is known to be important for somatosensory discrimination^57^. Since tactile discrimination is not affected by manipulation of STT inputs onto VPL (Fig. 5h,i), it then could be regulated by other inputs. One candidate input could be from the dorsal column nucleus (DCN), which integrate tactile information directly from low-threshold mechanoreceptors and indirectly from the postsynaptic dorsal column neurons^57,60,61^. Indeed, we found that silencing of the excitatory DCN→VPL pathway abolished the preference to the sand paper floor or to object with novel textures (Fig. 5h,i, Extended Data Fig. 11e). Interestingly, silencing this pathway had no effect on nociceptive response and SNI-induced pain sensitization (Extended Data Fig. 10e). We thus reveal distinct role of the DCN and STT inputs onto the thalamus in tactile discrimination and injury-induced pain sensitization, respectively.

## Discussion

**Fig. 6:**
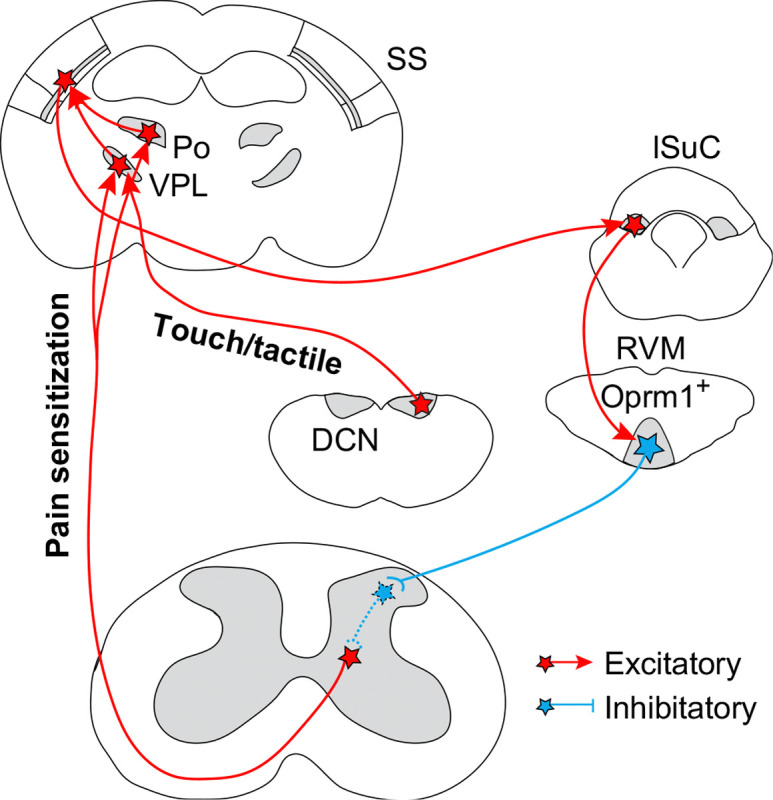
Circuit diagrams for touch and pain sensitization. Diagram of the long range circuit loop for mechanical sensitization and for touch/tactile sensation. Dash line indicates the hypothetical interneurons that connect the descending OPRM1^+^ inhibitory inputs to the excitatory STT pathways.

In conclusion, this study establishes the causal role of a multisynaptic pathway from spinal cord→thalamus (VPL + Po)→SS→lSuC→RVM→spinal cord that selectively mediates chronic mechanical pain after nerve injury or inflammation but is not involved in setting nociceptive thresholds in healthy mice (Fig. 6). Silencing any node within this long range circuit loop completely eliminates injury-induced mechanical hypersensitivity and restores normal mechanical responses in SNI mice. Conversely, repetitive activation of each node is sufficient to drive chronic mechanical hypersensitization in non-injured healthy mice. Thus, this long range circuit loop is necessary and sufficient for mechanical hypersensitization after injury or inflammation, rendering it a promising cellular target for treating chronic mechanical pain. Elucidating this core circuit loop for chronic pain lays the foundation for future studies investigating its interaction with the broader ‘pain matrix’ network.

Current models emphasize the important role of the PAG-RVM system in descending pain modulation. Our cTRIO tracing and *in situ* characterization revealed that more inhibitory than excitatory PAG neurons innervate OPRM1^+^ RVM^SC^ neurons. Silencing excitatory PAG terminals in the RVM had no effect on either nociceptive pain or injury-induced pain sensitization. Interestingly, a previous study using non-selective ablation of both excitatory and inhibitory PAG→RVM pathways also reported no effect on SNI-induced mechanical hypersensitivity^62^. Together, these two observations suggest that inhibitory PAG→RVM pathway also plays a limited role in pain sensitization. These results align with the well-documented analgesic effect of PAG stimulation, which may alleviate injury-induced pain sensitization via activation of inhibitory PAG input that suppress OPRM1^+^ RVM^SC^ neurons. PAG stimulation also suppress acute pain, an effect that may be mediated by OPRM1^−^ neurons in the RVM^10–12,63^.

Our results reveal the specific role of the STT in injury-induced pain sensitization, but not in nociceptive pain and discrimination of innocuous tactile stimuli. In contrast, the DCN→VPL pathway is specifically required for non-painful tactile discrimination, but is dispensable for both acute and chronic pain. Together, our findings support the model that distinct aspects of somatosensation are transmitted through separate ascending pathways (Fig.6), and highlight the need to identify ascending pathway(s) responsible for acute nociceptive pain. The spinoparabrachial pathway is a compelling candidate, given the important role of parabrachial nucleus in mediating the nocifensive responses to noxious stimuli^48,54,64^. Interestingly, activating either the STT→VPL→SS and STT→Po→SS pathway is sufficient to drive pain sensitization. However, silencing both pathway together is required to eliminate SNI-induced pain sensitization. These observations lead us to hypothesize that SNI sensitizes both STT→SS pathways, which operate in a redundant or parallel manner to initiate and maintain injury-induced pain sensitization.

Lastly, although we have delineated the anatomical and functional connections from the spinal cord to the brain, then came back to the spinal cord for chronic pain, the local spinal circuits connecting these two ends remains unresolved (Fig. 6). Given that OPRM1^+^ RVM^SC^ neurons are GABAergic and inhibitory, yet chronic mechanical pain requires persistent STT activation, we thus propose a plausible mechanism: these neurons may innervate a specific group of inhibitory interneurons that act as a ‘gate’, normally preventing non-painful somatosensory input from reaching the thalamus via the STT. Repetitive activation of OPRM1^+^ RVM^SC^ neurons could then induce long-term inhibition of this gating mechanism, permitting non-painful inputs to aberrantly activate the STT and drive chronic pain. A critical next step is to identify these inhibitory interneurons, which may constitute the long-sought gate circuit proposed by the ‘Gate Control Theory of Pain’ nearly sixty years ago^65,66^, and elucidate their connection to STT. In addition, we notice that cTRIO tracing identify the zona incerta, a region contains mostly inhibitory neurons, that directly innervates both OPRM1^+^ RVM^SC^ neurons and their presynaptic lSuC neurons (Fig. 3e, 4b). Activation of these inhibitory inputs should be able to suppress activity in both nodes of the descending pathway, thereby offering a powerful therapeutic target for alleviating chronic mechanical pain.

## Methods

### Mice

All procedures were in accordance with the US National Institutes of Health (NIH) guidelines for the care and use of laboratory animals and were approved by Stanford University’s Administrative Panel on Laboratory Animal Care. Mice (1.5 day-10 weeks) from both sexs were used in experiments. Genetically-engineered mouse lines used in this study included Oprm1^Cre/+^, Vglut2-IRES-Cre (JAX #016963). The Oprm1^Cre/+^ knock-in mouse line was generated in the Stanford University Transgenic, Knockout and Tumor model Center using conventional ES cell targeting strategies. The Cre recombinase cDNA, followed by the rabbit β-globin poly-A signal, was introduced via homologous recombination immediately after the start codon in exon 1 of the mouse *Oprm1* gene (Extended Data Fig. 1a). Heterozygous mice were generated by mating chimeric mice to C57BL/6 mice.

### Virus

The following viruses were produced and packaged in the lab and used in this study: AAV8^retro^-hSyn-H2BClover3-FLEX(LoxP)-H2BmRuby3 (2.0 E13 gc ml^−1^), AAV8^retro^-CAG-mCherry (5.0 E12 gc ml^−1^), AAV2^retro^-CAG-eGFP (5.0 E12 gc ml^−1^), AAV8-hSyn-FLEX(FRT)-mCherry (1.0 E13 gc ml^−1^), AAV8-hSyn-FLEX(FRT)-Clover3 (1.0 E13 gc ml^−1^), AAV8-hSyn-FLEX(FRT)-hM4D-IRES-EGFP (2.0 E13 gc ml^−1^), AAV8-hSyn-FLEX(FRT)-hM3D-EYFP (3.0 E13 gc ml^−1^), AAV8-hSyn-FLEX(FRT)-taCaspse3-TEVp (1.0 E13 gc ml^−1^), AAV8-hSyn-FLEX(LoxP)-Ruby3-FLEX(FRT)-Clover3 (1.0 E13 gc ml^−1^), AAV8^retro^-hSyn-FLEX-mTagBFP2-P2A-FlpO (5.0 E13 gc ml^−1^), AAV8^retro^-hSyn-mTagBFP2-P2A-Cre (5.0 E13 gc ml^−1^), AAV8-hSyn-FLEX(LoxP)-hM4D-IRES-GFP (5.0 E13 gc ml^−1^), AAV8-hSyn-FLEX(LoxP)-hM3D-IRES-mCherry (1.0 E13 gc ml^−1^), AAV8-hSyn-FLEX(LoxP)-hM3D-IRES- EGFP (1.0 E13 gc ml^−1^), AAV8-hSyn-FLEX(FRT)-jGCaMP7S (5.0 E13 gc ml^−1^), AAV1-hSyn-Cre (1.0 E13 gc ml^−1^), AAV8-hSyn-hM4D-mCherry (5.0 E12 gc ml^−1^), AAV8-hSyn-hM3D-mCherry (5.0 E12 gc ml^−1^), AAV8-EF1 α-FLEX(LoxP)-mScarlet (1.0 E13 gc ml^−1^), AAV8-Ef1a-DIO-hChR2(H134R)-EYFP (5.0 E12 gc ml^−1^), AAV8-hSyn-FLEX(FRT)-EGFP-P2A-TVA-T2A-oG (5.0 E13 gc ml^−1^). SADΔG-mCherry(EnvA) (2.0 E8 IU ml^−1^) was purchased from Salk viral core, CVS-N2cΔG-mCherry(EnvA) (5.0 E8 IU ml^−1^) was purchased from Jefferson Center for Vaccines. AAV5-Ef1a-DIO-hChR2(H134R)-mCherry (1.2 E13 gc ml^−1^, Addgene #20297) and AAV2-hSyn-DIO-hM4D(Gi)-mCherry (1.2 E13 gc ml-1, Addgene #44362) were purchased from Addgene.

### Surgery

#### Stereotaxic injection, optical fiber and cannular implantation.

Stereotaxic surgeries were performed on 5- to 7-week old mice under ketamine and xylazine (100 mg Kg^−1^ and 5 mg Kg^−1^, i.p.) anesthesia using a stereotaxic instrument (BenchMARK Digital, Leica). Virus was injected into the RVM (200 nl AAV, bregma −5.60 mm, lateral ±0.1 mm, ventral 5.75 mm), lSuC (200 nl AAV, bregma −3.45mm, lateral ±1.65 mm, ventral 2.40 mm), PAG (250 nl AAV, bregma −4.65 mm, lateral ±0.5 mm, ventral 3mm), SSp and SSs (200 nl AAV at site 1: bregma −1.45 mm, lateral ±3.75 mm, ventral −1.45 mm, 200 nl AAV at site 2: bregma −1.65 mm, lateral ±3.85 mm, ventral −1.55 mm), MOa (200 nl AAV at bregma +1.8 mm, lateral ±1.5 mm, ventral −1.35 mm), VPL (200 nl at bregma −1.8 mm, lateral ±2.0 mm, ventral 3.5 mm), PO (200 nl at bregma −1.8 mm, lateral ±1.6 mm, ventral 2.85 mm), DCN (70 nl per injection, at site 1: obex 0.0 mm, lateral ±0.6 mm, ventral −0.25 mm; site 2: obex 0.25 mm, lateral ±0.8 mm, ventral −0.3 mm; site 3: obex 0.5 mm, lateral ±1.1 mm, ventral −0.35 mm) with a pulled glass capillary at a slow rate (100 nl min^−1^) using a pressure microinjector (Micro 4 system, World Precision Instruments). The injection capillary was removed 5 min after the end of the injection. For mice used for terminal chemogenetic manipulation or fiber photometry, an infusion cannula (PlasticsOne) or optical fiber (Inper, HangZhou, China) was placed at least 200 μm above the target brain region and cemented to the skull using dental cement (Lang Dental Manufacturing). After surgery, a dummy cannula was insert and a cap was screwed on to keep the guide cannula from becoming occluded. Mice were allowed at least 2 weeks to recover and to express the virus before behavioral training commenced.

#### Spared nerve Injury.

SNI surgery were performed as previously described^28^ on 5- to 7-week old mice under ketamine and xylazine (100 mg Kg^−1^ and 5 mg Kg^−1^, i.p.) anesthesia. Briefly, following skin incision and blunt dissection to expose the sciatic nerve, and the tibial and common peroneal branches of the sciatic nerve were ligated with a 5.0 silk suture and transected distally, while the sural nerve was left intact. For sham surgery, only skin incision and blunt dissection were performed. After injury, skin was sutured, and mice were recovered on heated pad before being returned to their home cage. Mechanical and thermal thresholds were measured 2 days after the surgery.

#### CFA injection.

Mice were anesthetized with isoflurane (2%). 5 μl of CFA was injected into the plantar surface of the left hindpaw. Mechanical and thermal thresholds were measured at 1–3 days after CFA treatment.

#### Neonatal spinal cord injection.

The neonatal intraspinal cord injection method was modified based on the published method^14^. Neonatal pups were injected within 1.5–2.5 days after birth. The pups were covered in aluminum foil then surrounded in ice for 3–4 minutes until all movement stops and the skin color changes from pink to purple. 1–3 μl of rAAV vector containing 0.04% Trypan blue (for visualization of the injection site) was slowly injected into the spinal cord of cryoanesthetized neonates using 5 μl syringes (1 inch needle, 30 degrees bevel; Hamilton Company, Reno, NV). Only pups with clear visualization of blue line in the back of the body were used for experiments. After injection, pups were recovered on a warming blanket till the skin color changes to pink with body movements can be observed and then returned to the home cage. The neonatal injection did not lead to retrograde labeling extra neurons in the RVM (Extended Data Fig.2b,c).

#### Adult spinal cord injection.

Experiments in Fig. 5 were performed using adult spinal cord injection to avoid potential toxicity from long-term AAV1-Cre expression. A dorsal laminectomy was performed at vertebra level T12 to expose the spinal cord, followed by removal or the dura mater. 250 nl of AAV1-hSyn-Cre was injected in the spinal cord at 0.3–0.4 mm lateral to the central blood vessel, and 0.3 mm below the surface of the spinal cord. Four injections were performed on each side of the spinal cord at a space of 0.5 mm in the anterior to posterior direction.

### Fiber photometry

Fiber photometry experiments were performed at least 4 weeks after AAV8-hSyn-FLEX(FRT)-jGCaMP7S was injected into the RVM of the Oprm1^Cre/+^ mice receiving neonatal intraspinal cord injection of AAV8-retro-hSyn-FLEX(LoxP)-mTagBFP-P2A-FlpO, or after AAV8-hSyn-FLEX(FRT)-jGCaMP7S was injected into the lSuC of the vGlut2^Cre/+^ mice receiving RVM injection of AAV8-retro-hSyn-FLEX(LoxP)-mTagBFP-P2A-FlpO. The implanted fiber was connected to Fiber Optic Meter (FOM-02M, C-Light, SooChow, China) through an optical fiber patch cord (400 μm, 0.50 NA, Inper, Hangzhou, China). Mice were habituated for 3 days (30 min each) to fiber tethering before fiber photometry recording. To record fluorescence signals, a beam from a 480 nm LED was reflected with a dichroic mirror, and a lens connected to a CMOS detector (Thorlabs, Inc. DCC3240M) was used to focus the beam in order to record fluorescence data. At the patch cord’s tip, the LED power was less than 50 μW. A Labview program was used to control the CMOS camera to record the fluorescent signal. Using a RZ5D processor (Tucker-Davis Technologies), the analog voltage signal was digitalized, filtered (200 Hz low-pass), and sampled at 3 kHz. OpenEx software (Tucker-Davis Technologies) was used to record the fiber photometry data, and a custom MATLAB script (MathWorks) was used for data analysis. To record mechanical stimulation evoked response in uninjured and SNI mice, 4 g and 0.4 g von Frey fiber was applied to the lateral part of the plantar surface of the hind paw for 4–6 times with a 30 sec interval between each stimulation, respectively. For thermal stimulation, an infrared laser (70% of maximum power, Ugo Basile SRL) or dry ice was applied to the plantar surface through a glass plane underneath of the hind paw for 4–6 times with a 30 sec interval between each stimulation. For CFA injected group, 4 g (pre, and day 21 post CFA) and 0.4 g (day 2 and day 7 post CFA) von Frey fiber was applied to the plantar surface of the hind paw for 4–6 times with a 30 sec interval between each stimulation, respectively. For thermal stimulation, an infrared laser (70% of maximum power, Ugo Basile SRL) was applied to the plantar surface through a glass plane underneath of the hind paw for 4–6 times with a 30 sec interval between each stimulation. The video recording was synchronized with the fiber photometry recording through a TTL signal. The fluorescence change (ΔF/F) was calculated as (F-F_0_)/F_0_, where F_0_ is averaged fluorescence signals during 3 seconds baseline period before the onset of withdrawal reflex in each trial. The area under the curve (AUC) was calculated as integration of ΔF/F during each trial.

To assess the correlation between fiber photometry signals and locomotor speed, individual mouse was placed in a 25 × 45 cm arena. Locomotion bouts (4–6 per mouse) were identified, and the time course of each bout was normalized to a 0–100% scale. Data from a peri-event window (−50% to 150%) were extracted for analysis. The corresponding photometry signals and locomotor speeds were then obtained. A linear regression analysis was performed between the average photometry signal and the average locomotor speed during the first half of the locomotion bout (−50% to 50%) to quantify the relationship between neuronal activity and movement (spontaneous locomotion or pain-evoked escape).

### Chemogenetic Manipulation

For chemogenetic activation or silencing experiments, 0.1 mg Kg^−1^ clozapine were injected (i.p.) 30 mins before behavior tests. For terminal silencing experiments, 300 nl of 5 μM clozapine were infused into each target through the cannula 30 mins before behavior tests.

### Behavioral tasks

#### von Frey Test.

Each mouse was habituated in a red plastic cylinder on an elevated wire grid for at least 1 h prior to testing. Mechanical sensitivity was determined with a set of calibrated von Frey filaments (0.02 – 4 g, Ugo Basile). For SNI model, filament was applied to lateral part of the left hindpaw. Between individual measurements, von Frey filaments were applied at least 3s after the mice had returned to their initial resting state. A positive withdrawal response was defined as a brisk paw withdrawal, shaking, or licking directed at the stimulated paw. Movements related to general locomotion, weight shifting, grooming, or exploratory behavior were not considered withdrawal responses and were excluded from the analysis. The 50% paw withdrawal threshold was determined using the Dixon’s up-down method^24^.

#### Hargreaves Test.

Each mouse was habituated in a red plastic cylinder on a glass floor for at least 1 h prior to testing. A radiant heat beam (Hargreaves apparatus, Ugo Basile) was focused onto the left hind paw. The latency to hindpaw withdrawal was recorded with at least 2 trials per animal repeated ≥ 10 min apart. A cut-off latency of 20 s was set to avoid tissue damage.

#### Plantar Cold Test.

Each mouse was habituated in a red plastic cylinder on a glass floor for at least 1 h prior to testing. The test was conducted by applying a dry ice pellet to the plantar surface of the hind paw through a glass floor. The pellet was prepared by packing powdered dry ice into a modified syringe. The latency to paw withdrawal was recorded.

#### Formalin test.

eGFP or hM4D-expressing mice were infused with clozapine 30 min prior to test. 20 μl of 2.5% formalin was injected into the dorsal surface of the hind paw. Paw licking or biting duration were quantified during the phase I (0–10 min post formalin injection) and phase II (11–40 min post formalin injection).

#### Affective-Motivational Pain Behaviors.

Affective-motivational responses were evaluated as previously described^36^. Each mouse was habituated in a red plastic cylinder on a glass floor for at least 1 h prior to testing. Mice received a series of mechanical stimuli using von Frey filaments (0.07 g, light touch; 0.4 g, mild touch; 2.0 g, moderate touch). Each filament was applied ten times per session at 20–30 s intervals, with 60-s pauses between stimulus blocks. The time spent attending to the injured paw (intentional lifting or licking) and performing escape behaviors (accelerating away or rearing toward chamber openings) was quantified.

#### Spontaneous Pain Behaviors.

Spontaneous Pain Behaviors were evaluated as previously described^37^. Mice were habituated to the observation chamber for 3 days (30 min per day) before video recording. Mice were placed in a transparent plastic box (10×10×10 cm) on a glass floor. Spontaneous pain was quantified from 30-minute video recordings (bottom view). Flinching and licking behaviors were scored as 1 and 2, respectively; licking during grooming was excluded.

#### Mouse Facial Grimace Scale.

Facial grimacing was assessed as described^39^. Mice were habituated to the observation chamber for 3 days (30 min per day) before video recording. Briefly, each mouse was placed in a white wooden chamber (9×9×9 cm with a 2-cm floor extension) for an 8-minute video recording. Videos were scored online (https://painface.net) using a 0–2 scale for orbital tightening, ear position, whisker change, and nose bulge.

#### Airpuff-Evoked Blink Response.

The airpuff test was based on previously described^67^. Head-fixed mice were habituated on a running wheel for 3 days. The periocular area was recorded under infrared illumination. Compressed air (20 PSI) was delivered in 60-ms pulses every 3 seconds via polyethylene tubing positioned 2.5 mm from the right eye. Each trial consisted of 8 puffs, and blink responses were quantified from 3–4 consecutive trials, with ≥1 min between trials. Bonsai-rx software controlled simultaneous video recording and airpuff timing, and cropped the recording area.

#### Conditioned place aversion (CPA) test.

The CPA assay was used based on previously described^68^. Briefly, mice were habituated for 3 days (30 min each) to a custom-designed two-compartment CPA apparatus (30 cm × 25 cm × 20 cm) placed on an elevated mesh rack. Each chamber contained unique visual cues (black and grey stripes or plain grey walls). On the final day of habituation, baseline preferences were video-recorded for 10 min and movement was tracked using the custom tracking software running on MATLAB (MathWorks). Following baseline measurements, animals were confined to their preferred side of the chamber and paired with repeatedly stimulating the left hindpaw once every 10 s for 10 min using a 0.16 g filament. After pairing, mice were returned to their home cage for 20 min before re-exposed to the CPA chamber with free access to both side of the CPA chamber for 10 min. CPA scores were calculated by subtracting the time spent in the filament stimulation-paired side of the chamber during baseline from the time spent in the same side of the chamber during the re-exposure.

#### Open Field Test.

Each mouse was placed in a square arena (50×50 cm) with a defined center zone (30×30 cm). Locomotor activity and time spent in the center were recorded and analyzed as measures of exploratory drive and anxiety-like behavior.

#### Elevated Plus Maze (EPM).

The EPM consisted of two open and two enclosed arms (each 30 cm long), elevated above the floor. Mice were placed at the central junction facing an open arm and allowed to explore for 5 minutes. The time spent in the open arms was analyzed as an index of anxiety-like behavior.

#### Tail Suspension Test (TST).

Mice were suspended by the tail for 6 minutes in an enclosed chamber (55H × 15W × 11.5D cm). Total immobility time was analyzed.

#### Locomotion test.

Mice were habituated for 3 days (30 min each) to a custom-designed apparatus (50 cm × 50 cm × 30cm). On consecutive days, mice were injected (IP) with saline, clozapine (0.1 mg/kg, Cayman Chemical), or morphine (15 mg/kg, Hikma) and locomotor activity was measured for 10 min (for chemogenetic manipulation), or 30 min (for morphine-induced locomotion).

#### Textured novel object recognition test (NORT).

Textured NORT was used based on previously described^59^. Bilateral whisker plucking was performed 3 days prior to the habituation session. Mice were habituated to the behavior chamber for 10 mins 24 hours prior to the familiarization session. In the familiarization session, the mouse was placed in the chamber containing two copies of identical objects for 10 mins, and was then removed from the chamber and placed in a transport cage for 5 min. Next, the mouse was reintroduced to the chamber and presented with the familiar object and a novel object that only differ in texture. For both sessions, the experiments end when there has been a 20-sec physical interaction of both objects or when a 10 min period has been reached. The discrimination index was calculated as changes in the percentage of time spent on the selected object in each session.

#### Textured conditioned place preference (CPP).

Briefly, mice were habituated for 3 days (30 min each) to a custom-designed two-compartment CPA apparatus with unique visual cues in each compartment (30 cm × 25 cm × 20 cm). Baseline preferences were video-recorded for 10 min and movement was tracked using the custom tracking software running on MATLAB (MathWorks). Next, the floor of the less preferred side of the chamber was covered with fine grit sandpaper, while the other side was remained as the smooth plexiglass. For texture pairing, the animal was then reintroduced to the chamber, and the movement was video-recorded and tracked. 30 min after clozapine infusion, the animal was placed back into the chamber, and the difference of the time spent in sandpaper side during paring and test was calculated.

#### Measurement of heart rate and breathing rate.

The measurement of heart rate was performed as previously reported^69^. Briefly, after anesthetization with isoflurane, two electrodes were connected to the forelimb and hind limb of the mice. The electrocardiogram signal was filtered (10 Hz–500 Hz), amplified (×100) with an amplifier, digitized (10 kHz) and stored with Spike 2 software (Version 7.03). For breathing rate, the skin on top of the most posterior rib was tied and connected to a customized force transducer, which vibrate during each breathing cycle. The changes in voltage caused by the vibration of the force transducer were filtered (10 Hz–500 Hz), amplified (×100) with an amplifier, digitized (10 kHz) and stored with Spike 2 software (Version 7.03).

### Electrophysiology

Four weeks after AAV injection, mice (8–9 weeks old) were deeply anesthetized and cardially perfused with ice-cold cutting solution containing (in mM) 92 NMDG, 2.5 KCl, 1.25 NaH_2_PO_4_, 25 D-glucose, 20 HEPES, 2 thiourea, 5 Na-ascorbate, 3 Na-pyruvate, 30 NaHCO_3_, 12 N-acetyl-L-cysteine, 10 MgSO_4_, 0.5 CaCl_2_, and 0.005 NBQX (pH 7.2). Acute coronal slices (250 μm) containing RVM were made using vibratome (VT1000S, Leica), and recovered in cutting solution without NBQX, and then at least for 1 hr in aCSF containing (in mM) 125 NaCl, 2.5 KCl, 1.25 NaH_2_PO_4_, 10 D-glucose, 1 MgCl_2_, 26 NaHCO_3_, and 2 CaCl_2_ (pH 7.3) at room temperature. All slice recordings were conducted in room-temperature aCSF continuously perfused with O_2_ balanced with 5% CO_2_.

Whole cell patch-clamp recordings were obtained in RVM cells located adjacent to midline (<150 μm lateral from midline). For current clamp recording, glass microelectrodes (3–7 MΩ) were filled with internal solution containing (in mM) 130 K-gluconate, 1 KCl, 10 HEPES, 10 EGTA, 1 MgCl_2_, 1 CaCl_2_, 2 Mg-ATP, 0.2 Na-GTP (pH 7.3). Neurobiotin (0.3% w/v, Vector Laboratories) was also included to label recorded neurons. Descending RVM neurons were identified by expression of Clover3 under 40X water-immersion objective lens using fluorescent microscope (BX51WI, Olympus). Baseline firing rate was monitored for 3 min before and after application of drugs. DAMGO (1 μM, Tocris) was bath-applied with or without CTAP (1 μM, Cayman Chemical) for 10 min. Cells exhibited >20% variance in baseline firing rate were excluded from further analyses. Action potentials were detected and counted using Clampfit 10.2 software (Molecular Devices).

For voltage-clamp recordings, internal solution containing (in mM) 130 CsMeSO_3_, 1 MgCl_2_, 1 CaCl_2_, 11 EGTA, 10 HEPES, 2 Mg-ATP, 0.2 Na-GTP, and 5 QX314 (Tocris) was used. Picrotoxin (100 μM) was bath-applied throughout the recordings. RVM cells were held at −70 mV, and 2 ms long pulse of 470 nm blue light was emitted with LED-driver (M470L2, Thorlabs) every 15 sec to activate ChR2-expressing axon terminals. After monitoring baseline oEPSCs for 5 min, clozapine-N-oxide (5 μM, Cayman Chemical) was bath-applied for 15 min to activate hM4D. Amplitude of oEPSCs from baseline and the last 5 min of drug application were analyzed. All chemicals were obtained from Sigma unless specified otherwise.

After whole-cell patch clamp recording, slices were fixed with 4% paraformaldehyde in PBS at 4°C for overnight. Fixed slices were further sectioned to 50 μm using vibratome and washed with PBS for 3 times. Tissues were then incubated with streptavidin conjugated to Alexa Fluor 647 (1:1000, ThermoFisher) in PBS containing 0.5% Triton X-100 for overnight in room temperature. Sections were then washed with PBS for 3 times. Z-stack of confocal fluorescent images were taken with Airyscan2 (LSM980, Zeiss).

For experiments in Fig. 5b, AAV8-retro-mCherry was injected into lSuC and AAV8-ChR2 was injected into Po or VPL. Slices were prepared 4–5 weeks after virus injection. 2 ms long pulse of 470 nm blue light was emitted with LED-driver (M470L2, Thorlabs) every 15 sec to activate ChR2-expressing axon terminals.

### cTRIO tracing and analysis

Experiments in Fig. 3c,d,e and Fig. 4a,b were performed in Oprm1^Cre+/−^ mice with neonatal spinal cord injection of AAV8-retro-hSyn-FLEX(LoxP)-mTagBGFP-P2A-FlpO in the spinal cord, and Vglut2^Cre+/−^ mice with injection of AAV8-retro-hSyn-FLEX(LoxP)-mTagBGFP-P2A-FlpO in RVM, respectively. Two to six weeks later, 200 nl of AAV8-hSyn-FLEX(FRT)-EGFP-P2A-TVA-T2A-oG were injected into the RVM or lSuC using a stereotaxic instrument (BenchMARK Digital, Leica). Four weeks later, 200 nl SADΔG-mCherry(EnvA) or CVS-N2cΔG-mCherry(EnvA) was injected into the same area of the RVM or lSuC using the procedure described above. Mice were housed in a biosafety-level-2 (BSL2) facility for 7 days before sacrificing. For quantification of long-range input brain region, brain regions that are 1mm anterior or posterior to the injection site were excluded from analysis. Images were taken from consecutive 50 μm coronal sections using Zeiss Axioplan2 using 2.5x or 5x objective. Cell counting was performed manually using Fiji. For quantifications of subregions, boundaries were based on the Allen Institute’s reference atlas (https://mouse.brain-map.org/experiment/thumbnails/100048576?image_type=atlas). Potential double counting cells from consecutive sections was not adjusted. Fractional input was calculated by dividing the number of labeled neurons in each brain region by the total number of labeled neurons throughout the entire brain.

### Ablation of OPRM1^+^ RVM^SC^ neurons

AAV8-hSyn-FLEX(FRT)-taCaspse3-TEVp and AAV8-hSyn-FLEX(LoxP)-Ruby3-FLEX(FRT)-Clover3 was co-injected into the RVM of the Oprm1^Cre/+^ mice with neonatal injection of AAV8-retro-hSyn-FLEX-mTagBFP-P2A-FlpO (1 μl) in the spinal cord. For control mice, AAV8-hSyn-FLEX(LoxP)-Ruby3-FLEX(FRT)-Clover3 was injected into the RVM. The infected OPRM1^+^ RVM neurons express mRuby3 (red), and OPRM1^+^ RVM^SC^ neurons express both mRuby3 and Clover3 (yellow). Four weeks later, after behavioral test, the brains and spinal cord were collected from both groups for histological analysis.

### Immunostaining and RNAscope in situ hybridization

Mice were deeply anaesthetized with pentobarbital sodium solution and transcardially perfused with PBS, followed by 4% paraformaldehyde (PFA) in 1× PBS at room temperature. Brains and spinal cords were dissected from perfused mice and post-fixed in 4% PFA in 1× PBS at 4°C overnight, cryoprotected in 30% sucrose in 1× PBS at 4°C for overnight, embedded in OCT compound, and frozen using dry ice and kept at −80°C. Brains and spinal cords were cryosectioned (15 μm for RNAscope in situ hybridization or 50 μm for immunostaining) using a cryostat (Leica). For immunostaining, 50 μm sections were washed three times for 5 min each with 1× PBS. They were then incubated in a blocking solutions (0.3% PBST containing 10% normal donkey serum (LAMPIRE Biological Products 7332100)) for 2 h at room temperature. Subsequently, the section were incubated with primary antibodies diluted in 0.3% PBST containing 3% normal donkey serum overnight at 4°C. After three 10 min washes with PBS, the section were incubated with secondary antibodies diluted in 0.3% PBST containing 5% normal donkey serum for 2 h at room temperature. Following this, the sections were washed three times for 10 min each with PBS; Hoechst 33342 solution (ThermoFisher) was included in the second wash at 1:10,000 dilution. Finally, the sections were mounted with Fluoromount-G (Southern Biotech). Primary antibodies used in this study include rabbit anti-mCherry (1:1,000, 600401397, Rockland), rabbit anti-cFos (1:1,000, 226003, Synaptic systems), goat anti-TPH2 (1:500, ab121013, Abcam), mouse anti-HA (1:1000, 901514, Biolegend). Secondary antibodies included Alexa 594 or 647 conjugated donkey anti-rabbit antibodies, Alexa 647 conjugated donkey anti-mouse antibodies, and Alexa 647 conjugated anti-goat antibodies. All secondary antibodies were purchased from Life Technologies and used at 1:1000 dilutions. For RNAscope in situ hybridization, 15 μm sections were collected on glass slide, mRNA transcripts were detected using the RNAscope Fluorescent Multiplex Assay (Advanced Cell Diagnostics) and RNAscope Fluorescent Multiplex Reagent Kit v2 (cat. no. 323100). The RNAscope catalogue probes were used to detect Oprm1 (cat. no. 493251), vGat (cat. no. 319191), vGlut2 (cat. no. 319171), Tph2 (cat. no. 318691), Penk (cat. no. 318761) RNA molecules. Images were obtained using a Zeiss 710 confocal microscope using either 10× (Plan-Apochromat 10×, NA 0.45) or 20× (Plan-Apochromat 20×, NA 0.8) objectives. For imaging large spinal cord and brain sections, the tile-scan function was used and the tile images were stitched using Zeiss Zen microscope software.

For Fos Immunostaining, each SNI mouse was habituated in a red plastic cylinder on a glass floor for at least 1 h prior to testing. The left hindpaw were repeatedly stimulated once every 10 s for 10 min using a 0.16 g filament for the SNI mice whereas stimulated with 1.4 g filament for 90 times for 10 min in non-injured mice. Mice were perfused 1.5 h after the delivery of each stimulation, then processed for Fos immunohistochemical analysis.

### 2D registration of RVM descending neurons and 3D visualization

30–40 brain slices (50 μm) containing the RVM were scanned using Olympus VS120 for 2D registration. Custom MATLAB software were used to remove all image features outside the brain slices. Background subtraction and contrast enhancement of the Neurotrace 640 channel were then applied. The processed Neurotrace 640 images for each section were then serially analyzed using a combination of automated and manual methods. For a more detailed description of this procedure see Xiong et al., 2018^16^.

### TESOS imaging and single-cell tracing of OPRM1^+^ RVM^SC^ neurons

AAV8-EF1α-FLEX(LoxP)-mScarlet was injected into the brainstem of Oprm1^Cre/+^ mice (200 nl at bregma −5.6 mm, lateral ±0.1 mm, ventral 5.75 mm). 6 weeks post AAV injection, Mice were deeply anaesthetized with pentobarbital sodium solution and transcardially perfused with PBS, followed by 4% paraformaldehyde (PFA) in 1× PBS at room temperature. Brains and spinal cords were dissected and post-fixed in 4% PFA in 1× PBS at 4°C overnight. The brain and spinal tissues were then undergone through the TESOS clearing procedure through passive immersion, and the transparent embedding was performed 48 h post sample transparency as previously described^17^. Confocal images of transparently embedded samples were obtained using upright Nikon confocal microscope combined with a rotary microtome using the 40x objective, and reconstructed using custom software provided by Laboratory of Professor Hu Zhao at Chinese Institute for Brain Research, Beijing, China. Manual tracing of axons was performed using Lychnis (https://github.com/SMART-pipeline/Lychnis-tracing)^70^.

### Statistical analysis

No statistics methods were used to predetermine sample size. However, the sample sizes were comparable to those in prior publications. All analyses were performed using Prism (GraphPad software) and statistical methods were indicated when used. Normality was assessed for each dataset prior to analysis. Where data violated the assumption of normality, appropriate non-parametric tests were employed. When a t-test was used, it was performed as a two-tailed test. No method of randomization was used in any of the experiments. Experimenters were not blind to group allocation in behavioral experiments, but CPA score were measured automatically by custom tracking software running on MATLAB (MathWorks). All animals that completed the entire behavioral training and testing were included in the analysis. Unless otherwise indicated in the figure legend, data points were derived from individual mouse and are presented as mean ± SEM.

## Supplementary Material

Supplementary Files

This is a list of supplementary files associated with this preprint. Click to download.

• Statisticalanalysistable.docx

• Supplementalnote.docx

• QianclosedloopEDFigs.pdf

• RVMFiberphotometryvideo.avi

## Data Availability

Images showing the expression of OPRM1 in pain related regions in the brain, spinal cord and sensory neurons from the OPRM1^Cre^ X Ai14 mice are available at on Zenodo:https://zenodo.org/uploads/17110834?token=eyJhbGciOiJIUzUxMiJ9.eyJpZCI6IjVjNGU4ZWQ1LTViNjAtNDliNC1hN2Y1LTJjZjg0YjMyOGFmNyIsImRhdGEiOnt9LCJyYW5kb20iOiI2MWJmY2U4YTA3OWQ3OTgzNWU4ZDE1YTAwMjE0MDZlOCJ9.X_wmoi8TXd-kNrBi5RdofGVZeTF66ofo7S6Gfjh575x_xb77–97Xn0tV-avdF2BYhVrM8yxArErC63yVZR0xIA

## Code availability:

The code for tracking locomotion is available on GitHub using the link: https://github.com/thepenglab/posTracker.

All data and other materials used in the manuscript are available upon request to the corresponding author (xkchen@stanford.edu).
